# Cul4B promotes the progression of ovarian cancer by upregulating the expression of CDK2 and CyclinD1

**DOI:** 10.1186/s13048-020-00677-w

**Published:** 2020-07-04

**Authors:** Peng-jing Duan, Juan-hong Zhao, Li-li Xie

**Affiliations:** 1 Department of Gynaecology and Obstetrics, Affiliated Hospital of Shandong Medical College, 80 Jintan Road, Linyi, 276000 Shandong China; 2 Department of Gynaecology, The people’s hospital of Linshu, 182 West Shuhe Road, Linshu, 276700 Shandong China

**Keywords:** Cul4B, Ovarian cancer, CDK2, CyclinD1

## Abstract

**Background:**

Ovarian cancer is one of the most common malignant tumors in the female reproductive system with the highest mortality rate. Cul4B participates in the oncogenesis and progression of several malignant tumors. However, the role of Cul4B in ovarian cancer has not been studied.

**Results:**

High expression of intratumor Cul4B was associated with poor patient survival. Cul4B expression was associated with FIGO stage and Cul4B was independent risk factor of ovarian cancer disease-free survival and overall survival. In vitro studies revealed that overexpression of Cul4B promoted tumor proliferation while knockdown of Cul4B significantly inhibited the proliferation capacity of ovarian cancer cells. Mechanistically, Cul4B was found to promotes cell entering S phase from G0/G1 phase by regulating the expression of CDK2 and CyclinD1. Cul4B regulates the expression of CDK2 and CyclinD1 by repressing miR-372.

**Conclusions:**

The results revealed that high expression of Cul4B is associated with poor ovarian cancer prognosis and Cul4B may serve as a potential treating target for an adjuvant therapy.

## Introduction

Ovarian cancer is one of the most common malignant tumors in the female reproductive system [1]. The incidence rate of ovarian cancer ranks third while the mortality rate ranks first among all kinds of gynecological malignant tumors [2]. The 5-year survival rates of ovarian cancer in stages III and IV are less than 40 and 20%, respectively, which is a serious threat to women’s health [3]. The early stage symptoms of ovarian cancer are insidious, and metastasis often occurs before the symptoms. Most of the patients are in advanced stage of disease once diagnosed. The clinical treatments of ovarian cancer are mainly surgery and adjuvant therapies including chemotherapy, radiotherapy and biological treatment [4–7]. In recent years, with the development of molecular biology technology, gene therapy of ovarian cancer has become the focus of attention and shows a good application prospect [8, 9].

Cul4B (Cullin 4b), as a scaffold protein, participates in the formation of E3 ubiquitin ligase complex [10]. It regulates many important cellular activities including cell cycle, cell signal transduction, gene transcription and regulates individual growth and development [11]. Cul4B regulates cell proliferation and senescence by ubiquitination induced degradation of p53 and Cyclin E [12, 13]. It also upregulates the expression of p16, p21, PTGDS, mirRNA-371/372 at epigenetic level by regulating the acetylation and methylation of gene promoter by ubiquitination of H2A protein [14]. Cul4B also plays an important role in tumorigenesis. Cul4B inhibits the expression of Wnt antagonistic factors, PTEN and IGFBP3 by single ubiquitination of H2AK119 [15]. In bladder cancer [16], liver cancer [17] and non-small cell lung cancer [18], Cul4B plays the role of oncogene by inhibiting the expression of tumor suppressor gene. Cul4B has different functions in the oncogenesis and progression of tumor. However, up to now, the role of Cul4B in ovarian cancer has not been reported.

The purpose of this study is to investigate the role of Cul4B in the progression of ovarian cancer. First, the expression of Cul4B in human ovarian cancer was detected by immunohistochemistry. The results showed that the expression of Cul4B in human ovarian cancer was significantly associated with patient disease-free survival and overall survival. The overexpression and knockdown cell lines of Cul4B were then established to examine its potential role in the regulating of tumor malignant behaviors. CCK8 assay revealed that overexpression of Cul4B promotes tumor proliferation while knockdown of Cul4B inhibits tumor proliferation. Mechanistically, Cul4B was found to promotes cell entering S phase from G0/G1 phase by regulating the expression of CDK2 and CyclinD1.

## Results

### Cul4B expression is upregulated in ovarian cancer and high expression of Cul4B in the intratumor tissue is associated with poor patient prognosis

**Fig. 1:**
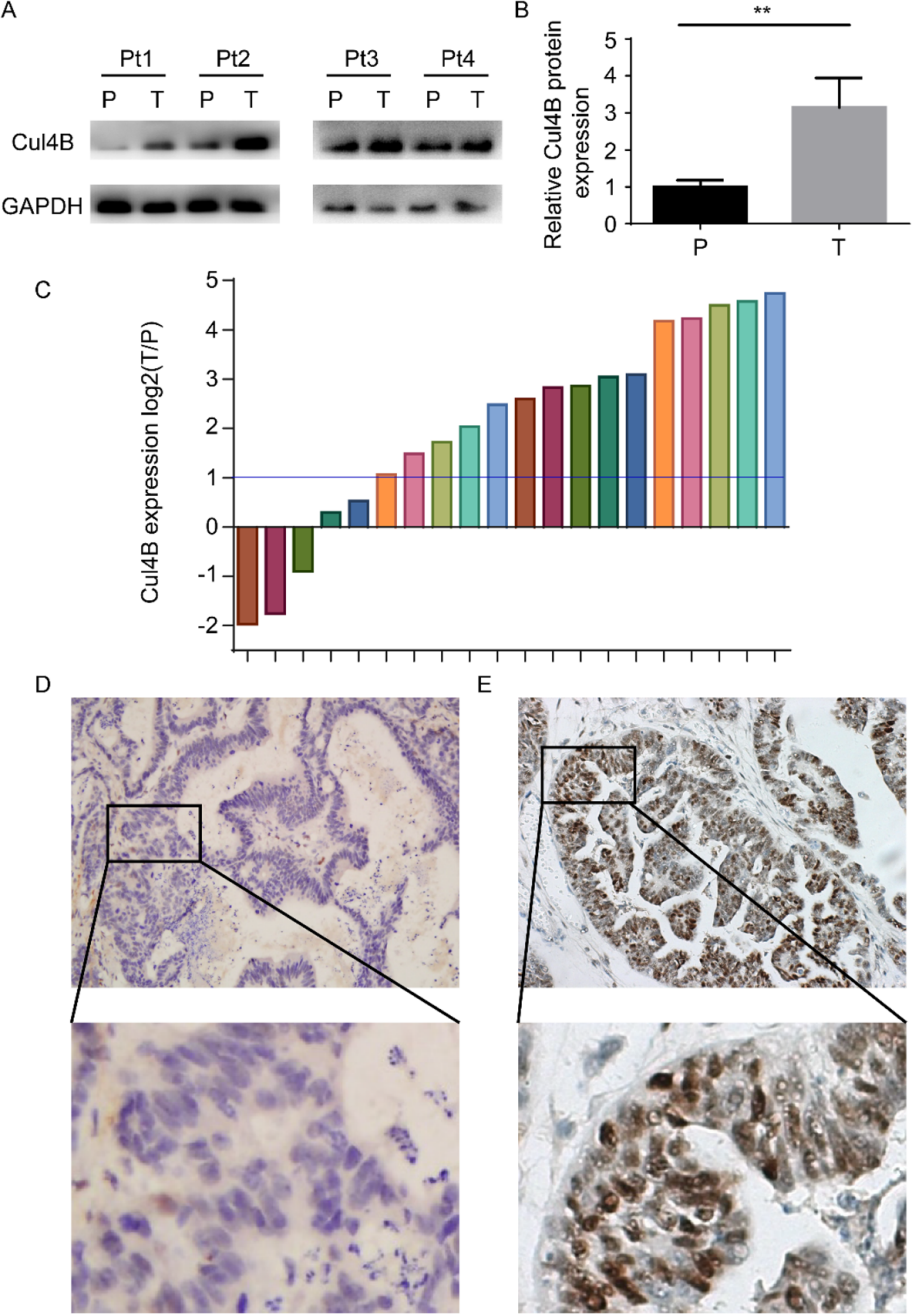
Western blot, qPCR and immunohistochemistry staining were performed to examine the expression of Cul4B in ovarian cancer tissues and adjacent normal tissues. **a** Western blot analysis of Cul4B expression in 4 paired tumor and adjacent normal tissues. **b** Statistical analysis of Western blot analysis results showed in (**a**). **c** qPCR analysis of Cul4B expression in 20 tumor tissues and paired normal tissues. **d**-**e** Typical pictures of intratumoral Cul4B expression classified as (**d**) relatively low or (**e**) relatively high according to the total positive staining area. Upper: magnification, ×50, lower: magnification, ×200

**Fig. 2:**
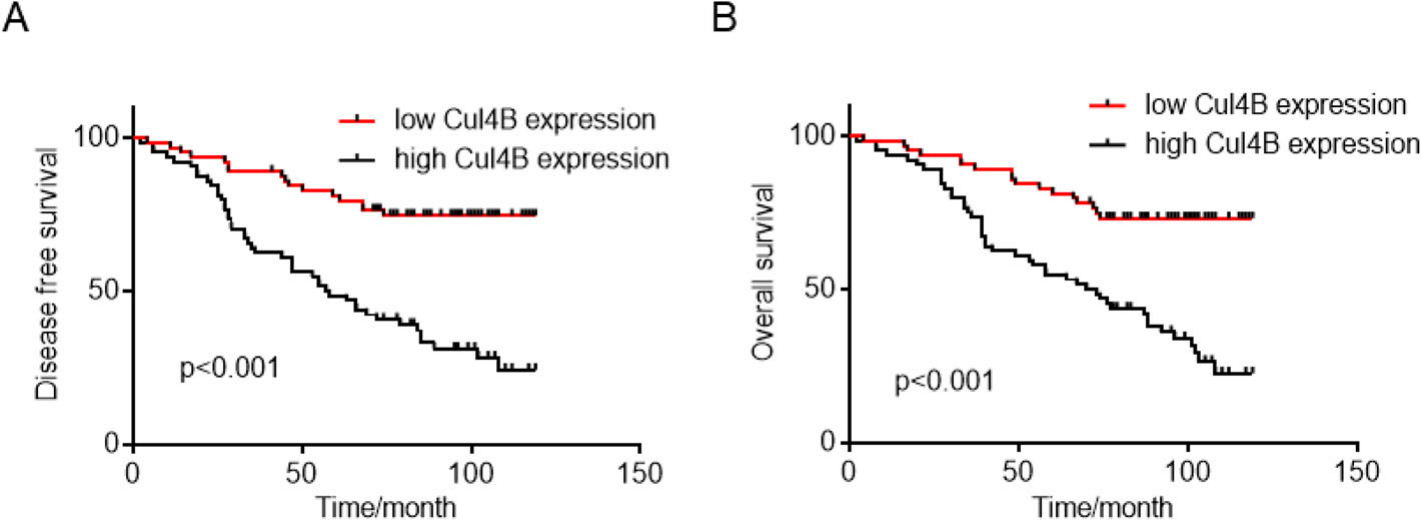
Analyses of impacts of Cul4B expression on the disease-free survival and overall survival of ovarian cancer patients. The disease-free survival (**a**) and overall survival (**b**) curves were plotted by Kaplan-Meier analysis and assessed by log-rank test

We first examined the expression of Cul4B in 4 tumor tissue and paired non-tumor tissues. Western blot analysis revealed that Cul4B expression is upregulated in tumor tissues (Fig. 1a-b). Real time qPCR analysis was also performed in 20 paired fresh tumor tissues and non-tumor tissues. Cul4B is upregulated in 15 (75%) patients (Fig. 1c). There is no report on whether expression of Cul4B in the intratumor tissue affects ovarian cancer patient survival, we analyzed the expression of Cul4B in the tumor tissue by immunohistochemistry staining analysis. Tumor tissues from 128 patients who underwent surgical resection in our hospital from January 2008 to January 2012 were obtained constructs into tissue microarray. As shown in Fig. 1d and e, expression of Cul4B can be divided into two groups namely low expression group (Fig. 1d; *n* = 64) and high expression group (Fig. 1e; n = 64). Cul4B was mainly expressed in the nucleus which is in accordance with previous studies. Basic clinicopathological parameters which may affect the survival of ovarian cancer patients including age, pathological type, tumor grade, tumor size, the International Federation of Gynecology and Obstetrics (FIGO) stage and CA125 were collected and analyzed. As shown in Fig. 2, patients with high expression of Cul4B are associated with poor recurrence free survival (Fig. 2a, *p* < 0.001) and poor overall survival (Fig. 2b, p < 0.001). We then analyzed the expression of Cul4B correlates with known prognostic factors by chi square analysis. The results revealed that intratumor Cul4B expression was associated with FIGO stage (Table 1). Univariate and multivariate analysis revealed that tumor grade and intratumor Cul4B expression are independent risk factors of patient disease-free survival while tumor grade, FIGO stage and intratumor Cul4B expression are identified as independent risk factors of patient overall survival (Table 2).

**Table 1:** Relationship between patient intratumor Cul4B and clinicopathologic features

Variable	Intratumor Cul4B		
	Low expression (*n* = 64)	High expression (*n* = 64)	p
Age (years)			
< 50	13	21	0.109
≥50	51	43	
Pathological type			
Non-serous tumour	37	32	0.375
Serous tumour	27	32	
Tumor grade			
1	10	9	0.804
2–3	54	55	
Tumor size			
< 10 cm	24	25	0.856
≥10 cm	40	39	
FIGO			
I-II	50	39	0.035
III-IV	14	25	
CA125			
< 70 U/L	27	29	0.581
≥70 U/L	36	35

**Table 2:** Univariate and multivariate analysis of factors associated with disease-free survival and overall survival rates of ovarian cancer following surgical resection

	Disease-free survival				Overall survival			
Clinicopathological factors	Univariate P	Multivariate			Univariate P	Multivariate		
		Hazard ratio	95% CI	p		Hazard ratio	95% CI	p
Age < 50 vs ≥50	0.034				0.049			
Non-serous tumour vs Serous tumour	0.279				0.386			
Grade 1 vs Grade 2–3	0.042	2.485	0.994–6.211	0.052	0.032	2.798	1.116–7.013	0.028
Tumor size < 10 cm vs ≥10 cm	0.244				0.212			
FIGO I-II vs III-IV	0.028				0.016	1.718	1.018–2.900	0.043
CA125 < 70 U/L vs ≥70 U/L	0.544				0.642			
Intratumoral Cul4B expression	0.000	3.797	2.142–6.733	0.000	0.000	3.240	1.844–5.692	0.000

### Overexpression of Cul4B promotes the proliferation capacity of ovarian cancer while knockdown of Cul4B inhibits the proliferation capacity of ovarian cancer

**Fig. 3:**
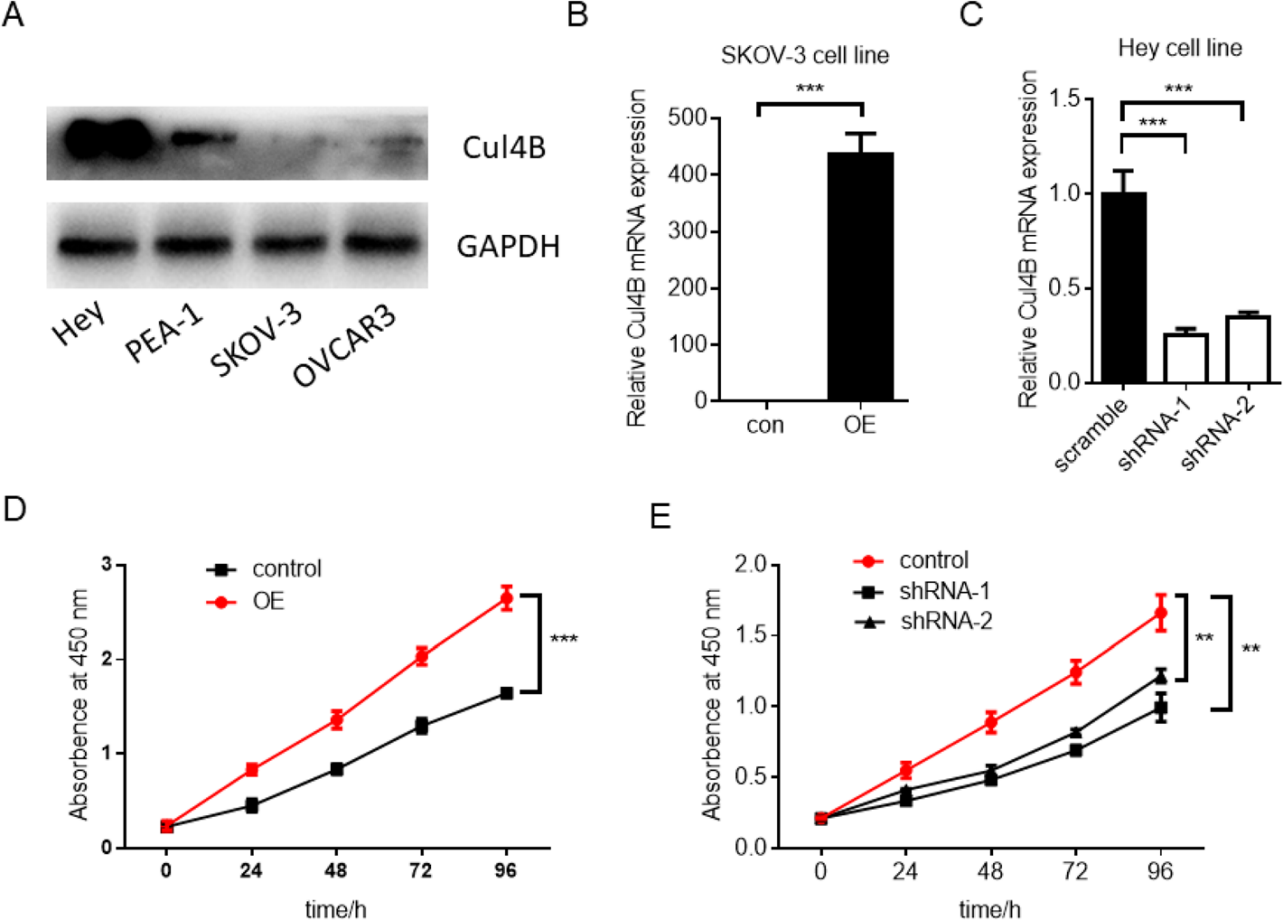
Expression of Cul4B in ovarian cancer cell lines and effects of overexpression or knockdown of Cul4B on the proliferation capacity of ovarian cancer cells. **a** Western blot analysis of Cul4B expression level in Hey, PEA-1, SKOV-3 and OVCAR3 cell lines. **b** Validation of Cul4B overexpression in SKOV-3 cell line by real-time qPCR analysis. **c** Validation of Cul4B knockdown efficacy in Hey cell line by real-time qPCR analysis. **d** Cell proliferation capacity was examined in control and Cul4B overexpressing SKOV-3 cell lines by CCK8 assay. **e** Cell proliferation capacity was examined in control and Cul4B knockdown Hey cell lines by CCK8 assay. (**: *p* < 0.01; ***: *p* < 0.001)

To further analyze whether expression of Clu4B in the ovarian tumor cells impact on its biological behavior, a series of ovarian cancer cell lines including Hey, PEA-1, SKOV-3 and OVCAR3 were collected and the expression of Cul4B in these cells were analyzed by Western blot. Expression of Cul4B in Hey cell line was relatively high and expression in SKOV-3 was relatively low (Fig. 3a). We therefore constructed overexpression cell line in SKOV-3 and knockdown cell line in Hey. The efficiency of overexpression and knockdown was validated by qPCR (Fig. 3b and c). CCK8 assay was then performed in these cell lines to analyze the potential effect of Cul4B on cell proliferation. The results revealed that overexpression of Cul4B promoted cell proliferation (Fig. 3d) while knockdown of Cul4B significantly inhibited the proliferation ability (Fig. 3e).

### Overexpression of Cul4B promotes ovarian cancer cells entering S phase from G0/G1 phase and upregulates the cell cycle regulating protein including CDK2 and CyclinD1

**Fig. 4:**
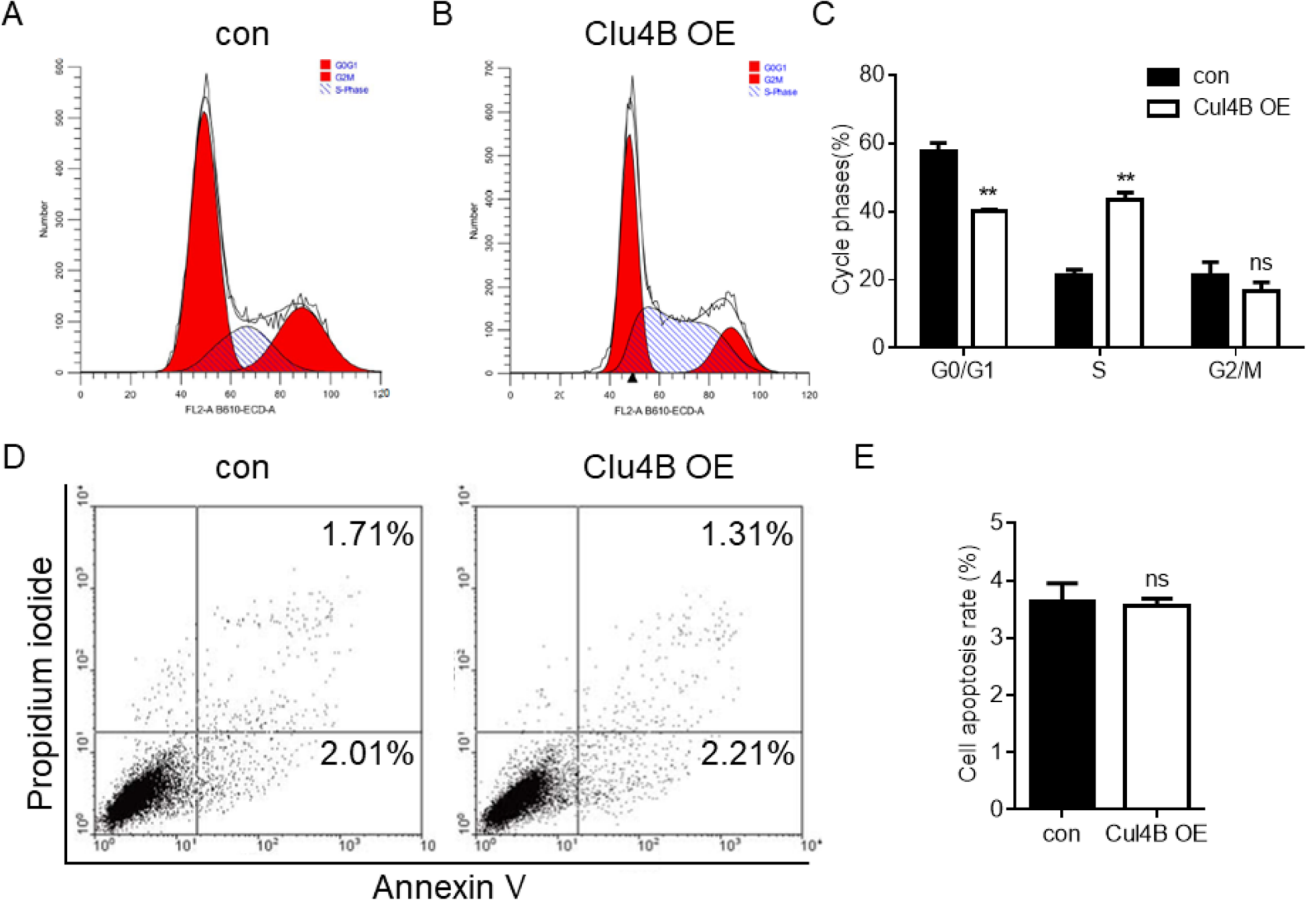
Cul4B promotes ovarian cancer cells entering S phase from G0/G1 phase while not affecting cell apoptosis. **a**-**b** Typical cell cycle analysis of con (**a**) and Cul4B overexpressing (**b**) cells. **c** Statistical analysis of phase proportion of G0/G1, S and G2 phase. **d** Typical cell death analysis of con (left) and Cul4B overexpressing (right) cells. **e** Statistical analysis of cell apoptosis rate of con and Cul4B overexpressing cells

**Fig. 5:**
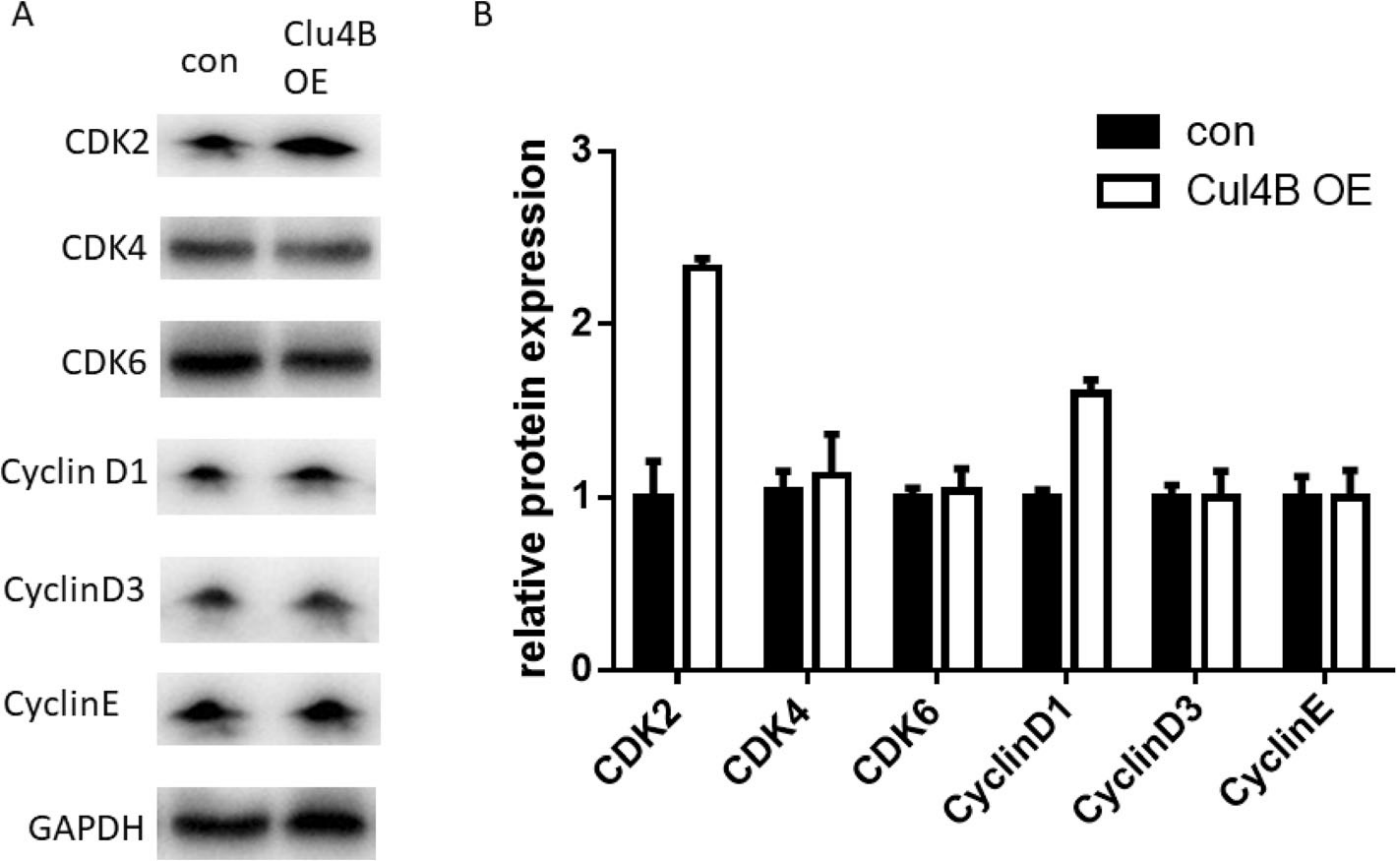
Overexpression of Cul4B upregulates the expression of CDK2 and Cyclin D1. **a** Blots of cell cycle related protein including CDK2, CDK4, CDK6, CyclinD1, CyclinD3 and CyclinE. **b** Quantification of expression of CDK2, CDK4, CDK6, CyclinD1, CyclinD3 and CyclinE by Image J analysis

As Cul4B exhibits regulating capacity on cell proliferation, we therefore set about exploring the potential underlying mechanism. We first examined cell cycle pattern and performed cell death analysis in control and Cul4B overexpressing ovarian cancer cells. As shown in Fig. 4a-c, Cul4B overexpression reduced the proportion of G0/G1 phase and increased the proportion of S phase, these results revealed that Cul4B promoted ovarian cancer cells entering S phase from G0/G1 phase. Cell apoptosis analysis reveal no different in cell apoptosis between control cells and Cul4B overexpressing cells which means that Cul4B does not affect ovarian cancer cell apoptosis (Fig. 4d-e). Cell cycle regulating proteins was believed to the most important molecule affecting cell proliferation. We examined whether overexpression of Cul4B really alters the expression of cell cycle checkpoint proteins including CDK2, CDK4, CDK6, CyclinD1, CyclinD3 and CyclinE. Western blot analysis and subsequent quantification revealed that overexpression of Cul4B significantly upregulated the expression of CDK2 and CyclinD1 (Fig. 5a and b). These results suggest that Cul4B regulates cell proliferation by upregulating the expression of CDK2 and CyclinD1 in ovarian cancer.

### Cul4B regulates the expression of CDK2 and CyclinD1 by repressing miR-372

**Fig. 6:**
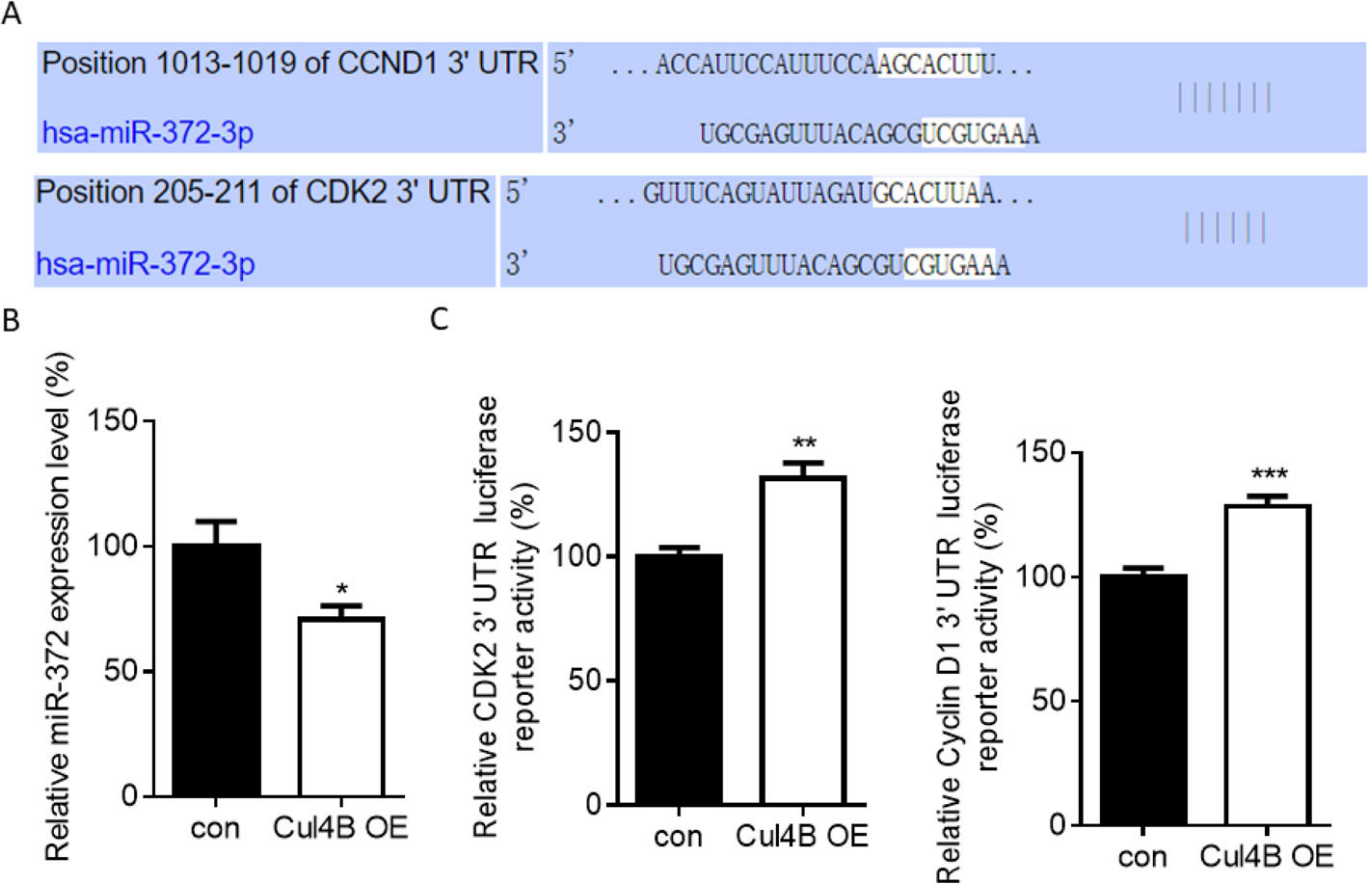
Cul4B regulated the expression of CDK2/CyclinD1 by repressing mir-372 and upregulating the 3′ UTR luciferase reporter activity of CDK2 and CyclinD1. **a** targetScan results showed that both 3′ UTR of CyclinD1 and CDK2 contains binding region for miR-372. **b** qPCR results revealed that Cul4B overexpression in ovarian cancer cells downregulates the expression of miR-372. c Luciferase reporter assay revealed that Cul4B overexpression in ovarian cancer cells upregulates the 3′ UTR luciferase reporter activity of both CDK2 and Cyclin D1

Previous study has revealed that Cul4B can upregulates the expression of CDK2 by repressing miR-372 [19]. We then examined whether Cul4B upregulates the expression of CDK2 and CyclinD1 by repressing miR-372 in ovarian cancer. We first searched targetScan to figure out whether miR-372 could also regulates the expression of CyclinD1. The results revealed that the 3′ UTR region of CyclinD1 also contains a binding position for miR-372 which means that CyclinD1 could be regulated by miR-372(Fig. 6a). We also examined whether Cul4B regulates the expression of miR-372 in ovarian cancer. We found that overexpressing Cul4B downregulates the expression of miR-372(Fig. 6b). We then examined the findings in Figure R4A by measuring the 3′ UTR luciferase reporter activity of CDK2 and CyclinD1. The results revealed that 3′ UTR luciferase reporter activity of CDK2 and CyclinD1 was upregulated in Cul4B overexpressing ovarian cancer cells (Fig. 6c). These results revealed that Cul4B regulated the expression of CDK2/CyclinD1 by repressing mir-372 and upregulating the 3′ UTR luciferase reporter activity of CDK2 and CyclinD1.

## Discussion

According to the cancer statistical data published in the CA: a cancer journal for clinicians in 2015, the annual incidence of ovarian cancer in China is 52,100 cases and the annual death is 22,500 cases, which is the highest among the three major gynecological malignant tumors [20]. The etiology of ovarian cancer is not clear. Genetic factors, environment factors, endocrine factors, age, fertility and mental factors may play a role in its pathogenesis [21, 22]. According to its clinicopathological and genetic characteristics, ovarian cancer can be divided into serous cancer, mucinous cancer, clear cell like cancer and endometrial like cancer, of which 70 to 80% are epithelial cancer [23]. At present, therapies of ovarian cancer include operation (oophorectomy/cytoreductive surgery), combined chemotherapy (paclitaxel/ carboplatin intravenous chemotherapy), targeted therapy (PARP inhibitor/ bevacizumab) and hormone therapy (goserellin/tamoxifen) [24, 25]. However, for the treatment of advanced ovarian cancer, there has been a lack of effective drugs for years [26]. With the breakthrough of innovative therapy, ovarian cancer treatment also ushered in new progress which emphasis on the significance of new target discovery in the treatment of ovarian cancer [27–29].

Cullin protein is a kind of relatively conservative protein family in eukaryotes [30]. Since the identification of the first Cullin member cul1 in 1996, many Cullin members have been found and identified [31]. Up to now, seven members have been found in human including cul1, cul2, Cul3, Cul4A, Cul4B, cul5 and cul7 [30]. They are all involved in the formation of different E3 ligase complexes. Recently, more and more studies show that Cul4B plays an important role in the development of tumor [32]. Cul4B promotes the proliferation and migration of tumor cells by ubiquitination of H2AK119 and transcription inhibition complex PRC2 [14]. Cul4B can inhibit the expression of Wnt/β pathway antagonists and activate Wnt/β-catenin signaling pathway to promote the development of liver cancer [33]. Cul4B was highly expressed in protein and mRNA levels in colon cancer tissues and was significantly related to the infiltration depth of tumor, lymph node metastasis, distal metastasis, tissue differentiation, vascular invasion and tumor pathological grade [34]. Moreover, patients with Cul4B positive tumor samples had higher recurrence rate and lower survival rate than those with negative tumor samples. Inhibiting the expression of CUL4B can inhibit the proliferation of colon cancer cells and increase the proportion of apoptosis [35]. As a proto oncogene, Cul4B plays an important role in the occurrence and development of various cancers [36, 37]. However, the role of CUL4B in ovarian cancer has not been reported.

In this study, we used immunohistochemistry to detect the expression of CUL4B in ovarian cancer and used the ovarian cancer cell lines as a model to analyze the effect of Cul4B on the proliferation of ovarian cancer cells. In accordance with previous studies, our results revealed that Cul4B was meanly expressed in the nucleus [38]. Expression of Cul4B affects patients’ prognosis and was independent prognostic factor of patient survival. Overexpression of Cul4B in SKOV-3 cell line promotes cell proliferation while knockdown of Cul4B in Hey cell line inhibits cell proliferation which is in accordance with findings in liver cancer and colon cancer. Mechanistically, Cul4B was found to regulate cell cycle by regulating the expression of CDK2 and CyclinD1. These results indicate a role of Cul4B in the regulation of cell cycle in ovarian cancer.

## Conclusions

Expression of Cul4B affects patients’ prognosis and was independent prognostic factor of patient survival. Overexpression of Cul4B promotes cell proliferation while knockdown of Cul4B inhibits cell proliferation. Mechanistically, Cul4B was found to promotes cell entering S phase from G0/G1 phase by regulating the expression of CDK2 and CyclinD1. Cul4B regulates the expression of CDK2 and CyclinD1 by repressing miR-372.These results indicate a role of Cul4B in the regulation of cell cycle in ovarian cancer.

## Materials and methods

### Patients

This study was approved by the Ethic Committee of Affiliated Hospital of Shandong Medical College. Written informed consents were obtained from all enrolled patients. Tumor tissues from 128 patients who underwent surgical resection in our hospital from January 2008 to January 2012 were obtained and formalin-fixed immediately after the resection of the tissue and then paraffin-embedded. These tissues were then applied for the construction of tissue microarray. The patients with FIGO stage over Ic at diagnosis or tumor relapsed after initial curative resection received chemotherapy. All study participants underwent followed-up ranged from 2 to 119 months. Fresh tumor tissues were obtained from January 2019 to January 2020 and the tissues were subjected to liquid nitrogen immediately after resection and stored at − 80 °C. The pathological type of the experiment samples used in this study were confirmed by two senior pathologists.

### Immunohistochemistry (IHC) staining

IHC staining for Cul4B was performed as previously described [39]. To be brief, sections were dried at 70 °C for 1 h and deparaffinized by using xylene and rehydrated by using alcohol gradients. Subsequently, sections were put into pH 6.0 citrate buffer to do microwave antigen retrieval. After that, the samples were blocked with goat serum, and incubated with the mouse anti-human Cul4B antibody (1:100 dilution; Cat. No. MABC556; Sigma) for at least 14 h at 4 °C. Finally, the specimens were washed with PBS solution and incubated with HRP-conjugated IgG and 3,3′-diaminobenzidine substrates to visualize the expression level. Expression of Cul4B in the tumor tissues was judged by two experienced pathologists and the H-score method was performed to quantify the expression of Cul4B. The H-score method was performed by combining the immunoreaction intensity and proportion of tumor cell staining. The score was calculated according to the following formula: (% cells of 1 + intensity score × 1) + (% cells of 2 + intensity score × 2) + (% cells of 3 + intensity score × 3). Two groups including high and low were created based on the histological scores.

### Western blotting

Western blotting was performed as previously described [40]. Total protein was extracted with RIPA lysis buffer with protease inhibitors and phosphatase inhibitors from ovarian cancer cell lines and tumor or adjacent normal tissues. A total of 30 μg protein were separated using 12% SDS-PAGE gel at 120 V for 90 min and electro-transferred onto polyvinylidene difluoride membranes at 300 mA for 2 h. Membranes were blocked with 5% BSA and then incubated with the primary antibody. Mouse antibody for GAPDH was purchased from Zsbio (Beijing, China). The mouse antibody for Cul4B were purchased from Sigma. The following antibodies were all from abcam: CDK2(ab32147), CDK4(ab108357), CDK6(ab124821), CyclinD1(ab16663), CyclinD3(ab183338), CyclinE (ab33911). Quantification of the blots was performed using the ImageJ software.

### qPCR

qPCR was performed according to the manufacture’s manual and the report previously described [41]. Total mRNA was isolated from the cells by using Trizol reagent according to the standard instructions. The total mRNA was then reversely transcribed into cDNA by using a reverse transcription kit (RR047A, Takara, Japan). Subsequently, Real-time quantitative PCR was conducted by using the SYBR Master Mix (Yeasen, China). GAPDH was selected as the housekeeping gene, and the presented primers were used:

*Cul4B* -Forward: 5′- ACTCCTCCTTTACAACCCAGG − 3′.

*Cul4B* -Reverse: 5′- TCTTCGCATCAAACCCTACAAAC -3′.

*GAPDH*-Forward: 5′- TGTGGGCATCAATGGATTTGG-3′.

*GAPDH*-Reverse: 5′- ACACCATGTATTCCGGGTCAAT-3′.

The sequences of these primers were from primerbank (https://pga.mgh.harvard.edu/primerbank/).

The levels of miRNAs were measured as previously described [19] and performed by using an All-in-One miRNA Q-PCR detection kit (GeneCopoeia, Inc.) according to the manufacturer’s protocol. snRNA U6 was used as the endogenous control. Each reaction was run in triplicate and in parallel. All primers used for miRNA qPCR were from GeneCopoeia, Inc.

### Cell culture and transfection

The ovarian cell lines Hey, PEA-1, SKOV-3 and OVCAR3 were purchased from the American Type Culture Collection (ATCC, Rockville, USA). Overexpression and knockdown cell lines were constructed by lentivirus induced transfection. The target sequence of shRNA-1 was 5′-CAATCTCCTTGTTTCAGAA-3′ and 5′-GAACTTCCGAGACAGACCT-3′ for shRNA-2.

### Cell proliferation assay

CCK8 assay were conducted to evaluate cell proliferation ability. Briefly, 100 μl cell suspension (3 × 10^3^ cells per well) were seeded in 96-well plates and then cultured at 37 °C, 5% CO_2_ for different days. At the same interval, the medium was discarded and the CCK8 solution was added to each well and incubated with cells for 2 h at 37 °C followed by measuring the absorbance at OD 450 nm with the Bio-rad microplate reader.

### Flow cytometry

Flow cytometry was performed as previously described [42]. For cell cycle analysis, cells were harvested and fixed in 70% ethanol overnight, stained with propidium iodide and RNase according to the manufacturer’s protocol, and analyzed via flow cytometry (BD Biosciences, San Jose, CA, USA). The data were analyzed with Modfit software (Verity Software House, Topsham, ME, USA). For apoptosis analysis, cells were washed with phosphate-buffered saline (PBS) and stained with annexin V and propidium iodide according to the manufacturer’s protocol (BD Pharmingen, San Diego, CA, USA). Fluorescence was measured using a FACSCalibur (BD Biosciences, San Jose, CA, USA) and analyzed using FlowJo (Tree Star, Ashland, OR, USA).

### Reporter constructs and luciferase assays

The pmir-GLO-CDK2 and Cyclin D1 3′-UTR vectors were generated by subcloning PCR-amplified full-length 3′-UTR of CDK2 and Cyclin D1 using HEK293 cDNA as a template into SacI–XhoI sites of pmir-GLO vector (Promega) downstream of the firefly luciferase gene.

For 3′-UTR reporter luciferase assays, cells were plated in 96-well plates and transfected with pmir-GLO reporter plasmids using Lipofectamine 3000 (Invitrogen). 24 h after transfection, luciferase assays were performed using the Dual-Luciferase Reporter Assay system (Promega) with a multilabel counter. Each firefly luciferase activity was normalized to Renilla luciferase activity of the pRL-TK reporter (cotransfected internal control). Transfections were performed in three independent experiments and assayed in quadruplicates.

### Statistical analysis

Statistical analyses were carried out by using the software SPSS version 25.0 and the survival analysis plots were drawn with Graphpad Prism 6. The relationship between clinical characteristics and the Cul4B expression were evaluated by Chi-square analysis. Prognostic factors were identified using the univariate and multivariate analysis. Kaplan–Meier method was used to plot the disease-free survival and overall survival curves of all enrolled ovarian cancer patients. Student’s t-test was used to analyze the results of in vitro experiments. *P* < 0.05 was considered statistically significant.

## Data Availability

The datasets used and/or analysed during the current study are available from the corresponding author on reasonable request.
